# Predictive Factors for Anastomotic Leakage after Laparoscopic and Open Total Gastrectomy: A Systematic Review

**DOI:** 10.3390/jcm11175022

**Published:** 2022-08-26

**Authors:** Umberto Bracale, Roberto Peltrini, Marcello De Luca, Mariangela Ilardi, Maria Michela Di Nuzzo, Alberto Sartori, Maurizio Sodo, Michele Danzi, Francesco Corcione, Carlo De Werra

**Affiliations:** 1 Department of Advanced Biomedical Sciences, Federico II University Hospital, 80131 Naples, Italy; 2 Department of Public Health, Federico II University Hospital, 80131 Naples, Italy; 3 Department of Surgery, San Valentino Montebelluna Hospital, 31044 Treviso, Italy

**Keywords:** anastomotic leakage, esophagojejunal anastomosis, total gastrectomy

## Abstract

The aim of this systematic review is to identify patient-related, perioperative and technical risk factors for esophago-jejunal anastomotic leakage (EJAL) in patients undergoing total gastrectomy for gastric cancer (GC). A comprehensive literature search of PubMed/MEDLINE, Embase and Scopus databases was performed. Studies providing factors predictive of EJAL by uni- and multivariate analysis or an estimate of association between EJAL and related risk factors were included. All studies were assessed for methodological quality, and a narrative synthesis of the results was performed. A total of 16 studies were included in the systematic review, with a total of 42,489 patients who underwent gastrectomy with esophago-jejunal anastomosis. Age, BMI, impaired respiratory function, prognostic nutritional index (PNI), alcohol consumption, chronic renal failure, diabetes and mixed-type histology were identified as patient-related risk factors for EJAL at multivariate analysis. Likewise, among operative factors, laparoscopic approach, anastomosis type, additional organ resection, blood loss, intraoperative time and surgeon experience were found to be predictive factors for the development of EJAL. In clinical setting, we are able to identify several risk factors for EJAL. This can improve the recognition of higher-risk patients and their outcomes.

## 1. Introduction

Anastomotic leakage (AL) is a major issue after esophago-jejunostomy (EJS). More generally, AL represents one of the most feared complications following any type of gastrointestinal anastomosis due to increased risk of morbidity and mortality as well as consequence on functional and oncologic outcomes [1]. It is defined as all conditions characterized by clinical or radiologic features of anastomotic dehiscence in accordance with the United Kingdom Surgical Infection Study Group [2,3,4].

Surgical technique, technology and perioperative management have evolved over time. Furthermore, the laparoscopic approach is often considered the standard of care in several abdominal diseases providing better short-term postoperative outcomes with no detrimental effects on oncological outcomes, and it has gained wide acceptance for surgical therapy of gastric cancer (GC) [5,6,7]. A recent meta-analysis of 2015 [8] regarding the anastomotic complications of EJS after total gastrectomy (TG) reported a similar rate between open and laparoscopic approaches (2.1% and 3.0%, respectively).

However, the etiology of AL is considered multifactorial. The leak rates after TG seems to be correlated firstly to the anatomic location of the anastomosis; EJS seems to be affected by a higher leak rate than gastrojejunostomy (GJ). Many factors (patient-related, perioperative as well as technical ones) have been identified as potential risk factors for esophago-jejunal anastomosis leakage (EJAL). In some instances, conclusive recommendations can be drawn, whereas others are still a matter of debate. The aim of this systematic review was to evaluate the current literature in order to identify patient-related, perioperative and technical risk factors for EJAL in patients undergoing TG for GC.

## 2. Materials and Methods

### 2.1. Search Strategy

A systematic review was carried out according to the guidelines of the Preferred Reporting Items for Systematic Reviews and Meta-Analyses (PRISMA) statement [9]. A comprehensive literature search of PubMed/MEDLINE, Embase and Scopus databases was carried out to identify articles published from 2003 until July 2022 to identify studies investigating risk factors for AL after TG.

A combination of the following keywords: ‘esophagojejunostomy’, ‘esophagojejunal anastomosis’, ‘total gastrectomy’, ‘anastomotic leakage’, ‘anastomotic leak’, ‘dehiscence’, ‘anastomotic complication’, ‘risk factor’, ‘predictive factor’, ‘predictor’, were used separated by the Boolean operators.

The literature search was reviewed independently by two authors (UB and MDL), first by title and abstract, to identify potentially relevant studies for full review. References of selected articles and relevant reviews were screened for potentially relevant articles. Only English language articles were evaluated.

### 2.2. Study Selection

Studies eligible for inclusion were full-text articles following inclusion criteria: (a) original articles; (b) patients who underwent gastrectomy with EJS; (c) patients evaluated which clinicopathologic or surgery-related factors predictive of AL by uni- and multivariate analysis; (d) provided an estimate of association between AL and related risk factors. Each paper was evaluated separately by two different researchers (UB and MDL). A third researcher was consulted in case of disagreement. Study protocols, case reports, reviews and meta-analyses were excluded. Furthermore, papers that dealt with complications of anastomoses in general (leakage, stenosis and bleeding), as well as papers showing the risk factors for leakage of all anastomoses (Bill-Roth I, Bill-Roth II, esophago-jejunal) without making a subgroup analysis, were excluded. Both randomized and non-randomized studies were included in the review.

### 2.3. Data Extraction

Data from the articles included in the present review were collected using predefined Microsoft Excel tables. For each study, the following data were extracted: year of publication, country, period of recruitment, study design, sample size, surgical approach, surgery intervention, method of esophago-jejunostomy, overall leak rate and esophago-jejunal leakage risk factor. Finally, a narrative synthesis was chosen as a way to illustrate the results.

### 2.4. Assessment of the Methodological Quality of Studies

All studies were assessed for methodological quality. For randomized studies, the validated.

JADAD score was chosen to assess the quality of randomized controlled trial RCTs collected, and every trial with a value ≥ 3 was included in the analysis [10]. MINORS scores were used for non-RCT studies. A threshold of ≥ 10 for non-comparative studies and ≥ 14 for comparative studies was set as inclusion criteria for the analysis [11].

## 3. Results

### 3.1. Search Strategy and Quality Assessment

**Figure 1:**
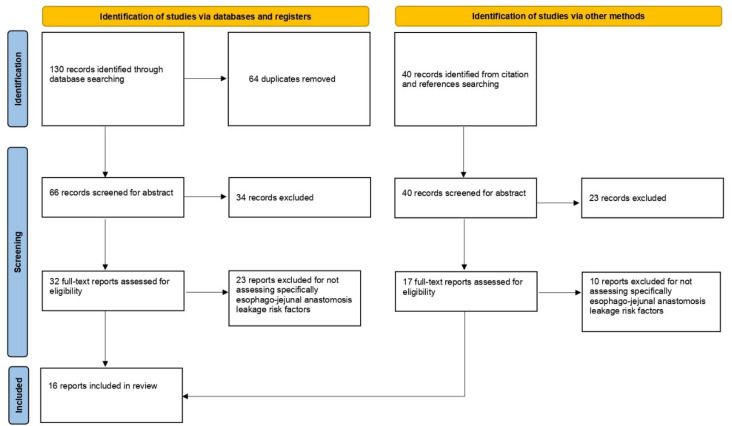
Prisma flow chart.

A total of 130 articles resulted from the literature search. Of these, 64 were removed because they were duplicated (PubMed/MEDLINE: 39; Embase: 48; Scopus: 43). Inclusion criteria of 66 articles were evaluated by reading only the abstract, and 34 were excluded because they did not respect them. Thus, 32 full-text articles were evaluated, and a further 23 articles were excluded for not specifically assessing the EJAL risk factors. Another 40 potential articles were identified through references cross search. Of these, 23 were excluded from reading the abstract for not respecting the inclusion criteria. The remaining 17 full-texts were evaluated, and 10 of them were excluded for not specifically assessing the EJAL risk factors. Finally, a total of 16 articles were included in the review (Figure 1).

JADAD Score was not used since no RCTs were found. All 16 studies were evaluated according to the MINORS score (Table 1). A threshold of ≥ 10 for non-comparative studies and ≥ 14 for comparative studies was set as inclusion criteria to the analysis. No study was eliminated for not meeting the threshold according to the MINORS score criteria.

There were 16 studies included in the analysis after quality assessment with a total sample size of 42,489 patients who underwent gastrectomy with EJS (Table 2).

### 3.2. Patients Related Factors

#### 3.2.1. Age

Two studies reported that age is an independent risk factor of EJAL in multivariate analysis [12,13]. Xing et al. [12] collected 390 patients and demonstrated that an age greater than 65 is closely related to the risk of developing anastomotic leakage (P: 0.043; OR: 3.882; 95% CI: 1.045–14.422). Kanaji et al. [13], in their prospective study with 185 patients, set 75 years old as the threshold to make the variable significant on anastomosis leak risk (P: 0.0097; OR: 7349; 95% CI: 1.63–39.475). They found a higher rate of positive leak tests in elderly than in younger (EJAL: 14.6 vs. 2.1%, *p* < 0.01). Similarly, Sierzega et al. [14] found that older age was associated with a greater risk of leakage (OR: 1.03, 95% CI: 1.00–1.05; *p* = 0.047). Even Takeuchi et al. [15] reported that age was a risk factor for anastomotic leakage. However, in a multivariate analysis, these results were not confirmed.

#### 3.2.2. Body Mass Index (BMI) and Obesity

Two studies reported that obese patients seem to have a greater risk of EJAL. Sugiyama et al. [16], in their retrospective analysis on 215 patients, show that BMI > 25 is significantly related to esophago-jejunal fistula (p: 0.0012; OR: 12.127; 95% CI: 2.652–72.933).

Similarly, Takeuchi et al. [15], comparing the results of a low-VFA (visceral fat area) group with a high VFA group, found a higher incidence of anastomotic leakage (*p* = 0.03) in this last group. In the multivariate analysis, they confirmed that high-VFA was identified as a predictor of anastomotic leakage (hazard ratio (HR): 4.62; 95% confidence interval (CI): 1.02–21.02; *p* = 0.048].

#### 3.2.3. Impaired Respiratory Function—American Society of Anesthesiology (ASA) Score

Three studies reported a correlation between impaired respiratory function and risk of EJAL. Trapani et al. [17], in a retrospective study on 1750 patients, reported a statistically significant correlation between respiratory comorbidities and a relevant risk factor for anastomotic fistula (OR: 2.27; P: 0.048). Similarly, Deguchi [18] found, in their multiple logistic regression analysis, that pulmonary insufficiency (OR: 3.300; 95% CI: 1.620–6.711) is an independent predictor of EJA leakage. Even Schietroma et al. [19] found that the risk of EJAL was 49% lower in the 80% FiO2 group (relative risk (RR): 0.61; 95% confidence interval (CI): 0.40–0.95) versus 30% FiO2. The risk of anastomotic leakage increased in male patients and in those with respiratory comorbidity (RR 1.93; 95% CI: 1.04–3.59; RR: 2.13; 95% CI: 1.01–4.42). This correlation confirmed only the percentage of inspired oxygen and preoperative respiratory disease in multivariate analysis. The risk of anastomotic dehiscence was reduced by 61% in patients assigned to 80% oxygen (RR: 0.46; 95% CI: 0.21–0.93; *p* = 0.05). Furthermore, patients with respiratory comorbidity had a 3.31-fold (95% CI: 1.22–9.10) greater probability of EJAL. Finally, they found that an ASA score of C3 was a risk factor for anastomosis integrity (odds ratio: 2.52; 95% CI: 1.5–4.3; *p* = 0.001).

#### 3.2.4. Preoperative Nutritional Status—Dysphagia and Gastric Stenosis

Only one study reports data about correlation between prognostic nutritional index (PNI) and EJAL risk [20]. The authors show that a PNI < 55 is statistically correlated with the risk of developing EJAL (P: 0.047; OR: 0.208; 95% CI: 0.044–0.981). Univariate analysis revealed that PNI significantly affected postoperative anastomotic leakage after laparoscopic TG (*p* = 0.039); the multivariate analysis using nine factors also confirmed it (OR: 0.208; 95% CI: 0.044–0.981, *p* = 0.047). Similarly, Sierzega et al. [14] found that the prevalence of anastomotic failure was also higher in patients with an Eastern Cooperative Oncology Group (ECOG) performance status of 2 or 3 (OR: 4·23, 95 percent CI: 2·06 to 8·83; *p* < 0.001). Subsequent regression analysis confirmed ECOG performance status of 2 or 3 (OR: 5.09, 95% CI: 2.29–11.32) as an independent risk factor for leakage.

Meyer et al. [21] found that the risk of EJS dehiscence was significantly associated with dysphagia and gastric stenosis. The anastomotic leak rate was 12.8% in patients with dysphagia and 4.9% in patients without dysphagia. Furthermore, they reported 16.7% of anastomotic leakage in patients with gastric stenosis, more than three times that of patients without stenosis.

#### 3.2.5. Alcohol Consumption, Diabetes and Chronic Renal Failure

Xing et al. [12] showed that alcohol consumption > 2 U/day is another risk factor for EJAL (P: 0.043; OR: 3.828; 95% CI: 1.043–14.050). This is the only study that reports alcohol as a risk factor significantly correlated to the EJAL [22].

Migita et al. [23] found that patients with HbA1c ≥ 7.0% had a higher rate of EJAL than those without it (23.1 vs. 5.1%; *p* < 0.05). This result, along with chronic renal failure (*p* < 0.01), was confirmed as an independent risk factor for EJAL in the multivariate analysis.

#### 3.2.6. Tumor Histology

Among selected studies, few data are available on the correlation between the risk of EJAL and tumor histology. Rawicz et al. [24], in their retrospective study of 114 patients, showed that gastric cancer with mixed-type histology is correlated with an increased risk of EJAL. The risk was significantly higher for the mixed-type compared to other histological GC types (OR: 12.45; 95% CI: 1.03–150.10; *p* = 0.0472; adjusted).

### 3.3. Operative Factors

#### 3.3.1. Laparoscopy

Kodera et al. [25] reported a significant difference in the incidence of EJAL between LTG and open gastrectomy (5.4% vs. 3.6%; *p* < 0.001). This data seems to be consistent with that of Trapani et al. [17]. They found that, in a series of patients between 2000 and 2018, compared with open conventional total gastrectomy, laparoscopic procedures seemed to increase the risk of EJAL (15.1% vs. 6.4%). Although a minimally invasive approach was implemented in four centers starting from 2009, considering only patients treated from 2009, the EJAL rate remained significantly higher after laparoscopic surgery than the open surgery group (15.1% versus 7.7%; *p* = 0.007).

Similarly, Sakamoto et al. [26] found that the analyses for each fiscal year showed higher anastomotic leakage in LTG than OTG, although the differences in some years were not significant. By analyzing a nationwide database of 58,689 patients and performing a propensity-score matching analysis, they showed an EJAL rate almost two-fold-increased after laparoscopic surgery (2.9% vs. 1.7%; *p* < 0.001).

**Table 1:** Minors score of the included articles.

Reference	Year	Country	Period of Recruitment	Study Design	N	Minors Score
Barchi et al. [27]	2019	Brazil	2009–2017	Retrospective	258	21
Çetin et al. [28]	2019	Turkey	2013–2016	Retrospective	80	18
Deguchi et al. [18]	2012	Japan	1999–2008	Retrospective	1640	10
Kanaji et al. [13]	2015	Japan	2008–2011	Prospective	185	17
Kodera et al. [25]	2019	Japan	2012–2013	Retrospective	1366	22
Meyer et al. [21]	2005	Germany	2002	Prospective	649	10
Migita et al. [23]	2012	Japan	2001–2011	Retrospective	327	12
Oshi et al. [20]	2018	Japan	2006–2014	Retrospective	131	18
Rawicz et al. [24]	2020	Poland	2016–2019	Retrospective	114	12
Sakamoto et al. [26]	2020	Tokyo	2012–2017	Retrospective	24,458	20
Schietroma et al. [19]	2013	Italy	2009–2012	Prospective	171	12
Sierzega et al. [14]	2010	Poland	1999–2004	Retrospective	690	12
Sugiyama et al. [16]	2017	Japan	2007–2014	Retrospective	215	16
Takeuchi et al. [15]	2016	Japan	2006–2015	Retrospective	65	12
Trapani et al. [17]	2020	Italy	2000–2018	Retrospective	1750	22
Xing et al. [12]	2021	China	2009–2019	Retrospective	390	18

**Table 2:** Characteristics of the included articles.

Reference	Surgical Approach	Surgery Intervention	Method of Esophagojejunostomy	Overall Leak Rate (%)	EJ Leakage Risk Factor Identified	Statistical Analysis
Barchi 2019 [27]	Open and laparoscopic	Completion gastrectomy: 50 Total gastrectomy: 208	End-to-side circular stapler Laparoscopic: side-to-side endolinear stapler	5.8	Completion gastrectomy	Uni- and multivariate analysis
Cetin 2019 [28]	Open	Total gastrectomy: 80	End-to-side circular stapler	16.2	Intraoperative time, additional organ resection	Uni- and multivariate analysis
Deguchi 2012 [18]	Open	Total gastrectomy: 1349 Proximal gastrectomy: 190 Completion gastrectomy: 101	End-to-side circular stapler	2.1	Older pt (>65 years), pulmonary insufficiency, D2 or D2+ dissection, additional organ resection, omentum resection, thoracotomy, intraoperative blood transfusion, operative time and postoperative creatinine level	Uni- and multivariate analysis
Kanaji 2015 [13]	Open	Total gastrectomy: 185	End-to-side circular stapler	4.8	Age ≥ 75, surgeon experienced <30 cases	Uni- and multivariate analysis
Kodera 2019 [25]	Open and laparoscopic	Total gastrectomy: 11,366	x	OpenStageI: 3,6 Laparoscopic StageI: 5,4 Open Stage II–IV: 3.6 Laparoscopic StageII–IV: 5.7	Laparoscopic approach	Comparison between matched cohorts
Meyer 2005 [21]	x	Total gastrectomy: 649	Stapler Hand sewing	5.5	Preoperative dysphagia, gastric stenosis, positive (metastatic) lymph nodes, nicotine abuse, multivisceral resection	Uni- and multivariate analysis
Migita 2012 [23]	Open and laparoscopic	Total gastrectomy: 317 proximal gastrectomy: 10	Circular stapler	5.8	HbA1c ≥ 7.0%, chronic renal failure, proximal gastrectomy, anastomotic troubles	Uni- and multivariate analysis
Oshi 2018 [20]	Laparoscopic	Total gastrectomy: 131	End-to-side circular stapler OrVil	9.9	PNI ^4^	Uni- and multivariate analysis
Rawicz 2020 [24]	Open and laparoscopic	Total gastrectomy and proximal gastrectomy: 114	x	4.6	Mixed histological type of GC	Uni- and multivariate analysis
Sakamoto 2020 [26]	Open and laparoscopic	Total gastrectomy: 24,458	x	Open 1,7; Laparoscopic: 2,9	Laparoscopic approach	Comparison between matched cohorts
Schietroma 2013 [19]	Open	Total gastrectomy: 171	Circular stapler Manual suture	14.6	Percentage of inspired oxygen, coexisting respiratory disease, ASA score ≥ 3, prolonged operative time	Uni- and multivariate analysis
Sierzega 2010 [14]	x	Total gastrectomy: 690	Circular stapler	5.9	Splenectomy, pancreatectomy, age, ECOG 2–3	Uni- and multivariate analysis
Sugiyama 2017 [16]	Laparoscopic	Total gastrectomy 215	FE-EA ^1^ Circular stapler	FE-EA: 2.0; Circular stapler: 8.8	BMI > 25, circular stapling anastomosis	Uni- and multivariate analysis
Takeuchi 2016 [15]	Open	Total gastrectomy: 75	x	H-VFA ^2^: 23,1 L-VFA ^3^: 6,1	H-VFA, age	Uni- and multivariate analysis
Trapani 2020 [17]	Open, laparoscopic and robotic	Total gastrectomy: 1750	End to Side Side to side Partially Mechanical Totally Mechanical	6.6	Respiratory disease	Uni- and multivariate analysis
Xing 2021 [12]	Open and laparoscopic	Total gastrectomy: 390	Circular stapler	2.6	Age > 65, Alcohol consumption of >2U/day	Uni- and multivariate analysis

^1^ FE-EA: functional end-to-end anastomosis; ^2^ H-VFA: high visceral fat area; ^3^ L-VFA: low visceral fat area; ^4^ PNI: prognostic nutritional index, x: missing data.

#### 3.3.2. Anastomosis Type

One study shows a relationship between the type of anastomosis and EJAL [16]. The authors retrospectively analyzed two types of anastomoses that they performed laparoscopically in 215 patients—intracorporeal reconstruction with a double or hemi-double stapling technique with a circular stapler with a transoral or transabdominal technique compared to intracorporeal reconstruction with a functional end-to-end anastomosis (FEEA). In multivariate analysis, circular anastomosis is a statistically significant risk factor of developing leakage (P: 0.0208; OR: 7.128; 95% CI: 1.347–47.277).

#### 3.3.3. Completion Gastrectomy

Another operative risk factor that correlates with a higher incidence of EJAL is completion gastrectomy. Barchi et al. [27] showed this significant correlation (OR: 3.34; 95% CI: 1.06–10.57; P: 0.040). Furthermore, Kanaji et al. [13] found that patients with a history of previous gastrectomy had a higher rate of positive leak tests (18.8 vs. 1.8%, *p* < 0.01).

#### 3.3.4. Additional Organ Resection—Extent of the Operation—Blood Loss

Although not confirmed by multivariate analysis, Deguchi’s study [18] showed that the extent of the operation, including lymph node dissection (*p* = 0.014), combined resection of other organs (*p* = 0.007), omental resection (*p* = 0.017), blood loss (*p* = 0.036) and intraoperative blood transfusion (*p* = 0.02), was significantly associated with anastomotic leak. Similarly, Migita et al. [23] confirmed that patients with EJAL also had greater blood loss than those without it (820 vs. 425 g, *p* < 0.05). They also found patients with macroscopic oesophageal invasion had a higher risk of EJAL than patients without it (15.8 vs. 4.5%, *p* < 0.05). In the study of Cetin et al. [28], it was confirmed that additional organ resection (*p* = 0.002) significantly increased the rate of EJAL. In addition, as demonstrated by multivariate analysis, organ resection is an independent risk factor for EJAL. In particular, splenectomy and pancreatectomy increased the risk of leakage [14]. In this study, subsequent regression analysis identified only splenectomy (OR: 2.58, 95% CI: 1.08- 6.13) as independent risk factor. Finally, even hyperthermic intraperitoneal chemotherapy (HIPEC) was identified as a potential risk of postoperative complications [24].

#### 3.3.5. Intraoperative Time

Cetin et al. [28] showed a correlation between the risk of EJAL and a longer intraoperative time (P: 0.032; OR: 10.416; 95% CI: 0.011–0.820) [28]. The results of the multivariate analysis revealed that operative time (*p* = 0.032, OR: 10.416, 95% CI: 0.011–0.820) is an independent risk factor for EJAL. Furthermore, Deguchi et al. [18] identified operative time (OR: 1.012; 95% CI: 1.007–1.018) as an independent predictor of EJAL in a multiple logistic regression analysis. Migita et al. [23] found that patients with EJAL had a significantly longer median operative time than those without it (330 vs. 290 min; *p* < 0.05). These results were also confirmed by Schietroma et al. [19], who found, in a multivariate analysis, a prolonged operative time (odds ratio 3.08; 95% CI 1.3–8.2; *p* = 0.02) as another factor significantly associated with a higher risk of EJAL.

#### 3.3.6. Surgeon Experience

The impact of surgeon experience was documented by Kanaji et al. [13]. In their study, the author showed a higher EJAL rate among less-experienced surgeons than by highly experienced surgeons (12.0 vs. 2.2%; *p* < 0.01). However, they showed that both surgeon groups had a relatively high EJAL rate in elderly patients.

## 4. Discussion

TG remains a challenge due to oncological and technical aspects, whether performed laparoscopically or not [29]. EJAL represents one of the most serious and potentially lethal complications after TG, with incidences ranging from 2.1% to 14.6% [18,19,23,30,31,32,33,34]. It has a negative impact on other postoperative outcomes and the need for re-operation by up to 61%, with a mortality of up to 50% [13,14,35].

However, prevention of EJAL remains a real challenge after total gastrectomy. The aim of our review is to analyze and report all the potential EJAL risk factors. We waived a pooled analysis because we included comparative and non-comparative studies, with different endpoints, intervention components and variable outcomes. In this setting, a non-negligible clinical and methodological heterogeneity was hypothesized that could significantly influence the quantitative analysis. Therefore, we provided a narrative synthesis to overcome this concern. We divided these into patient-related and operative factors.

Regarding the patient-related factors, age is a significant risk factor for intraoperative and postoperative anastomosis complications because elderly patients are more likely to have comorbidities that can alter their physiology, as well as a slowed healing ability [18].

Some studies reported a higher incidence of postoperative complications in older patients with advanced GC receiving neoadjuvant therapy (NAT) treated with a laparoscopic approach [7,36]. Owing to the poor ability of older patients to respond to stimuli, the early clinical symptoms of anastomotic leakage might be atypical and prone to be missed or misdiagnosed; thus, more attention should be paid to EJAL in older patients [12]. Among older patients, impaired respiratory function represents a common comorbidity that should affect EJAL risk. Actually, impaired respiratory function (common in European latitudes) has been mentioned among determinants for the differences in EJAL incidence between eastern and western countries [33,37]. Schietroma et al. [19] showed that the EJAL risk was 49% lower in patients treated by supplemental oxygen administration during, and 6 h after, open total gastrectomy. This aspect should furtherly focus the attention on preoperative setting, aiming to optimize the respiratory work-up before total gastrectomy, according to ERAS recommendations [38]. Similarly, although Isozaki et al. [30] demonstrated that impaired respiratory function had no impact on the EJAL rate, Haga et al. [39] proposed the estimation of physiologic ability and surgical stress (E-PASS) for a surgical audit in elective digestive surgery. Their findings showed that severe respiratory disease was one of the important preoperative risk factors in the E-PASS system. More generally, upper gastrointestinal surgery can have a negative effect on the inflation of the lungs because of the postoperative. The resulting hypoxia may delay the healing of the anastomosis.

Aging also affects preoperative nutritional status; as surgery increases among elderly patients, PNI is an important variable to be considered preoperatively [20].

In our review, we found the paper of Oshi et al. [20] that reported both univariate and multivariate analysis that PNI significantly affected EJAL rate. The advanced stage of the disease and patient-related morbidity can negatively affect the nutritional status. The role of preoperative nutritional support in improving postoperative outcomes of patients with gastric cancer is well documented [40,41]. Meyer et al. [21] proved that dysphagia and gastric stenosis were independent, significantly influencing factors for the occurrence of EJAL, with odds ratios of 3.408 and 3.762, respectively. They suggested that an adapted, short-term, hypercaloric preoperative nutritional supplementation [42] can improve the nutritional status prior to the procedure and can be supportive in the prevention of EJAL in patients with dysphagia and gastric stenosis.

Regarding the BMI, in both reported papers, the authors ascribe part of this result to the fact that obese patients are generally more demanding and, consequently, that the anastomosis fashioning requires even more skills regardless of type and approach.

Regarding alcohol consumption and diabetes, Xing et al. [12] showed that alcohol consumption > 2 U/day is another EJAL risk factor. Migita et al. [23] found that patients with HbA1c ≥ 7.0% had a higher rate of EJAL than those without it. However, alcohol consumption has previously been associated with increased postoperative complications in patients with colorectal cancer. Rullier et al. [43] and Sorensen et al. [44] reported that smoking and alcohol abuse were major risk factors for anastomotic leakage in colorectal surgery. Xing et al. [12] reported that alcoholism may affect the healing process and lead to impaired anastomotic integrity in various ways. For example, it may lead to increased perioperative bleeding because of bone marrow toxicity and decreased levels of fibrinogen, factor VII, and von Willebrand factor.

The negative impact of diabetes mellitus on both incisional wounds [45] and intestinal anastomosis [46] is well known, and the preoperative control of the blood glucose level may have a direct role in anastomosis healing [23]. Therefore, preoperative improvement in diabetes control is necessary in patients undergoing gastrectomy to reduce EJAL. This is consistent with data of other authors that reported diabetes mellitus as an independent factor for anastomotic leakage in colorectal anastomosis. Similarly, chronic renal failure is also associated with EJAL [23]. In these cases, accurate postoperative surveillance and multidisciplinary management are required.

Regarding the operative factors, we found that laparoscopy, type of anastomosis, completion of gastrectomy, additional organ resection—extent of the operation, blood loss, increased operative time and surgeon experience were independent risk factors for EJAL occurrence.

The benefits of the laparoscopic approach in the short-term outcomes have made it the gold standard in the surgical treatment of various gastrointestinal diseases [47,48]. However, laparoscopic TG remains a challenge due to oncological and technical aspects. Although a recent meta-analysis of 2015 [8] regarding the anastomotic complications of EJS after TG reported a similar rate between open and laparoscopic approaches, there is no doubt that the fashioning of an EJS represents one of the most critical procedural steps. Kodera et al. [25] reported a significant difference in the incidence of EJAL between laparoscopic TG and open gastrectomy (5.4% vs. 3.6%), as well as Trapani et al. [17]. Similarly, Sakamoto et al. [26] reported an EJAL rate almost two-fold-increased in the laparoscopic group (2.9% vs. 1.7%). Kodera and colleagues [25] highlighted that surgeons in Japan were reluctant to introduce laparoscopic TG as a routine practice for clinical Stage I cancer and were even more reluctant to perform laparoscopic TG for the advanced GC. In another article by Etoh et al. [49], comparing laparoscopic TG with Open TG using the NCD database, found no significant difference in the EJAL incidence between the two approaches (6.1% in open surgery vs. 5.3% in laparoscopic surgery, *p* = 0.59).

Regarding the anastomosis type, Sugiyama et al. [16] showed, with multivariate analysis, that circular anastomosis is a statistically significant risk factor for developing leakage. The authors justify this correlation with the fact that most of these patients had a BMI > 25 and that, therefore, anastomosis is created on fragile and soft tissue in a restricted operating field.

More generally, Kawamura et al. [50] reported that overlap esophagojejunostomy was safer than the OrVil procedure, especially in anastomotic stenosis, recommending this technique for anastomosis construction during laparoscopic TG due to a lower rate of postoperative complications. Similarly, Kosuga et al. [51] reported (in multivariate analysis) that the anastomotic procedure with the single-stapling technique was significantly associated with a lower rate of postoperative anastomotic complications than a hemi-double-stapling technique. The frequency of anastomotic leakage was lower in the modified group (3.1%) than in the original group (9.9%), although the difference was not statistically significant. However, EJAL is likely to result from intraoperative technical failures. For this reason, the rate of EJAL might decrease with the prevention and proper intraoperative management of an incomplete anastomosis.

Complex surgical procedures related to the occurrence of adhesions, such as completion gastrectomy or removing the gastric remnant, were considered risk factors for EJAL [27]. Completion gastrectomy is a more complex operation than a total gastrectomy because the removal of the remaining portion of the stomach is complicated by the presence of visceral adhesions due to the previous operation. In fact, it seems to be clear that this type of intervention has greater operating time, greater blood loss and, therefore, greater postoperative complications [27]. Many studies reported that the operative time was markedly longer in the EJAL group than in the group with no leakage, and it was found to be statistically significant by both univariate and multivariate analyses. More generally, they have also reported that prolonged operative time is related to morbidity after gastrectomy [52,53,54]. Many factors affect prolonged operative time. Procedural duration is generally prolonged in advanced tumor cases, but it does not always lead to EJAL. Spleen and pancreas resection increase the risk of postoperative complications. The risk of EJAL is closely related to the degree of complexity of the surgical intervention. The higher the stage of the tumor, the greater its degree of infiltration into the surrounding tissues. Although not confirmed by multivariate analysis, Deguchi et al. [18] found that the leakage rate was significantly associated with the extent of the operation, including lymph node dissection, combined resection of other organs and omental resection.

Finally, the role of the microbiome as a potential risk factor for AL has gained more evidence in lower than upper gastrointestinal surgery [55]. In fact, it is believed that the contamination of the suture line by the bacterial flora can favor infections and microabscesses and, therefore, local ischemia and risk of dehiscence. In a study of 55 patients who underwent esophagectomy, a significant difference was found in microbiota composition between preoperative saliva samples and intraoperative gastric mucosa samples in patients who developed anastomotic leakage [56]. In this context, some studies have proven the effectiveness of antibiotic application in the prevention of esophagojejunal anastomotic leakage after total gastrectomy [57,58]. However, clinical data remain poor and not conclusive in this regard.

## 5. Conclusions

In conclusion, our systematic review identified several risk factors for EJAL in patients who underwent total gastrectomy for GC. Although more prospective trials are needed, this study provides major insights into identifying higher-risk patients and improve their outcomes.

## Data Availability

The data presented in this study are available on request from the corresponding author.
